# 3D printed PLA Army-Navy retractors when used as linear retractors yield clinically acceptable tolerances

**DOI:** 10.1186/s41205-019-0053-z

**Published:** 2019-11-21

**Authors:** Joshua V. Chen, Alexis B. C. Dang, Carlin S. Lee, Alan B. C. Dang

**Affiliations:** 1 Department of Orthopaedic Surgery, University of California, San Francisco, CA USA; 2 Department of Surgery, Orthopaedic Section, San Francisco VA Health Center, San Francisco, CA USA

**Keywords:** 3D printing, Additive manufacturing, Medical devices, Surgical instruments, Optimization, Polylactic acid

## Abstract

**Background:**

Modern low-cost 3D printing technologies offer the promise of access to surgical tools in resource scarce areas, however optimal designs for manufacturing have not yet been established. We explore how the optimization of 3D printing parameters when manufacturing polylactic acid filament based Army-Navy retractors vastly increases the strength of retractors, and investigate sources of variability in retractor strength, material cost, printing time, and parameter limitations.

**Methods:**

Standard retractors were printed from various polylactic acid filament spools intra-manufacturer and inter-manufacturer to measure variability in retractor strength. Printing parameters were systematically varied to determine optimum printing parameters. These parameters include retractor width, thickness, infill percentage, infill geometry, perimeter number, and a reinforced joint design. Estimated retractor mass from computer models allows us to estimate material cost.

**Results:**

We found statistically significant differences in retractor strength between spools of the same manufacturer and between manufacturers. We determined the true strength optimized retractor to have 30% infill, 3 perimeters, 0.25 in. thickness, 0.75 in. width, and has “Triangle” infill geometry and reinforced joints, failing at more than 15X the threshold for clinically excessive retraction and costs $1.25 USD.

**Conclusions:**

The optimization of 3D printed Army-Navy retractors greatly improve the efficacy of this instrument and expedite the adoption of 3D printing technology in many diverse fields in medicine not necessarily limited to resource poor settings.

## Background

Current literature explores the role of 3D printing in changing the surgical landscape through the new ability to create more resource efficient in-house surgical instruments, along with anatomical models of patients that assist in training and preoperative planning, prosthetics, personalized surgical equipment better suited for patient morphologies, and 3D printed implants [1–14]. Notably, these vast strides in 3D printing technologies are also allowing healthcare providers to create robust, low-cost medical and surgical equipment in resource scarce areas, such as in developing nations and in aerospace medicine where weight and space limitations exist [15–17]. 3D printing allows for rapid prototyping and manufacturing that expedites the improvement of medical equipment, examples of which are the development of better splints, syringes, and surgical suction tips [18–20]. Furthermore, it is shown that the high heat used during the 3D printing process sterilizes the final print [21]. Specifically, 3D printed polylactic acid (PLA) Army-Navy retractors offer a cheaper, lighter-weight, and more space-efficient alternative to traditional stainless steel Army-Navy retractors without compromising surgical retraction capabilities [22]. Army-Navy retractors are commonly used in a myriad of procedures involving retraction of shallow incisions and are listed at an online retail price of approximately $24 USD per retractor. Though literature exists in quantifying the mechanical strength of 3D printed Army-Navy retractors, none have sought to optimize printing settings and retractor designs, which could lead to additional discussion about improving the efficacy of 3D printed surgical instruments and allow healthcare providers in resource scarce areas to adopt this technology.

## Methods

### Software and design

Army-Navy retractor models were designed in Autodesk® Fusion 360™ (Autodesk®, Inc.). Standard Tessellation Language (STL) files of these models were exported from Autodesk Fusion 360 and imported into Slic3r Prusa Edition – 1.38.7–prusa3d (Prusa Research), a software that processes STL files into thin slices that can then be converted into g-codes, a numerical control programming language that provides spatial instructions to the 3D printer to guide the printing process, ultimately building the print one layer at a time. All prints in this study have layer heights of 0.15 mm. Print settings were systematically varied in Slic3r Prusa Edition to produce unique g-codes and ultimately determine the most strength optimal print settings for our designed PLA Army-Navy retractors.

**Fig. 1:**
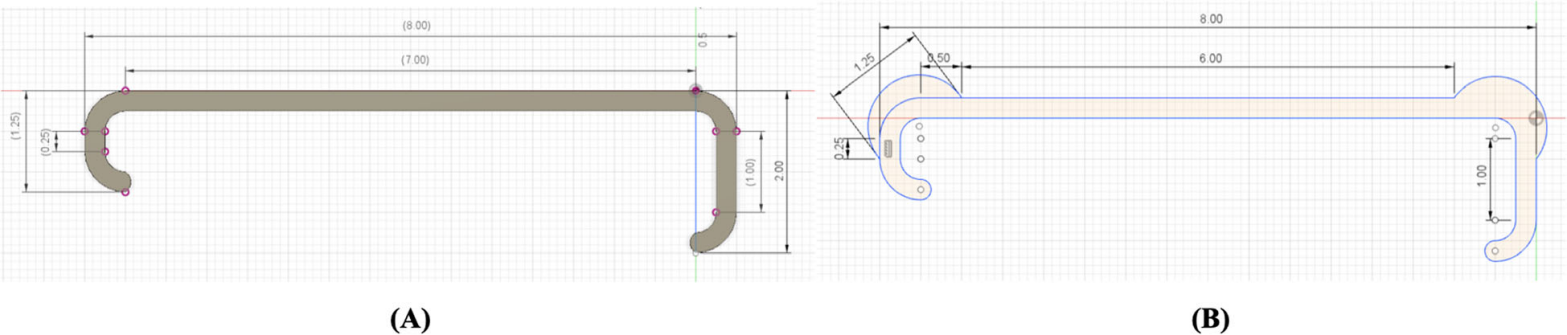
**a** Standard retractor design and **b** reinforced retractor design in inches. **c** The Army-Navy retractor is oriented on the print bed horizontally such that no support material is needed

Control retractors are set to have 20% infill, 2 perimeters, a “Grid” infill geometry, “Rectilinear” top/bottom fill pattern, default Slic3r Prusa Edition print settings, and are oriented horizontally so that no support material is needed (Fig. 1a, c). Infill geometry, infill percent, and perimeter number were changed, respectively, with all other parameters held constant in Slic3r Prusa Edition. For retractors with reinforced joints (Fig. 1b), differing retractor widths, and differing retractor thicknesses, separate retractor models were designed in Autodesk Fusion 360 and the corresponding STLs were imported separately into Slic3r.

### 3D printer model and hardware

G-codes are loaded into an SD card and inserted into the Original Prusa i3 MK3 3D printer (Prusa Research) where the print can be selected and started. An Original Prusa i3 MK3 kit is priced at $750 USD and a fully assembled Original Prusa i3 MK3 3D printer is priced at $1000 USD. A 0.4 mm nozzle was used with the extruder temperature set to 235 °C for all layers and bed temperature was set to 60 °C for all layers. Essentium engineering grade 1.75 mm PLA was used, priced at $50/kg.

### Mechanical testing

**Fig. 2:**
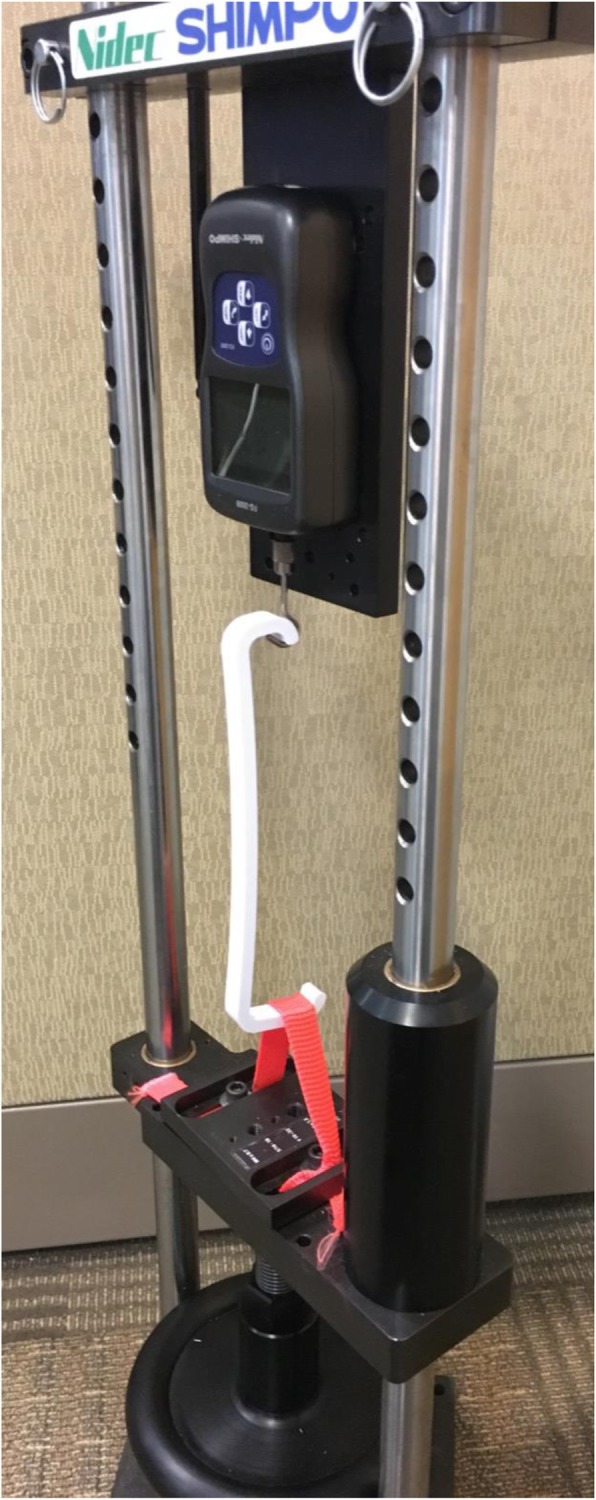
Nidec-Shimpo FGS-1000H hand wheel test stand and FG-3009 force gauge with oriented retractor

Mechanical testing was performed using a Nidec-Shimpo FGS-1000H hand wheel test stand and FG-3009 force gauge, where retractors were pulled until failure and the maximum force withstood was measured (Fig. 2). In this test stand, retractors were orientated so that the short arm of the retractor was placed on the force gauge hook and the long arm of the retractor was placed on the strap, such that the strap was as far away from the body of the retractor as possible to subject the retractors to similar torque.

Using the Nidec-Shimpo force gauge and test stand, a stainless steel Army-Navy retractor was used to determine appropriate retractor strength, with 35 N +/− 5 N indicated by expert surgeons as strong or clinically excessive retraction causing tissue tearing and damage, based upon feel of the retractor on the surgeons’ hands.

### Retractor sets

Four sets of control retractors were printed to test inter-spool and inter-manufacturer variability; three of these sets were printed using three separate Essentium PLA filament spools, and the remaining set was printed using MakerBot PLA filament (Table 1). The estimated mass of this retractor is 10.20 g, resulting in a material cost of $0.51 USD without machine expenses. The time to print these retractors is approximately 1 h 20 min. Two-tailed T-tests will be used to determine whether there exist statistically significant differences in retractor strength.

**Table 1:** Control retractor sets varied by spool and filament type

Set Number	Number of Retractors	Filament Type
No. 1	7	Essentium (Spool 1)
No. 2	5	Essentium (Spool 2)
No. 3	10	MakerBot PLA
No. 4	10	Essentium (Spool 3)

**Fig. 3:**
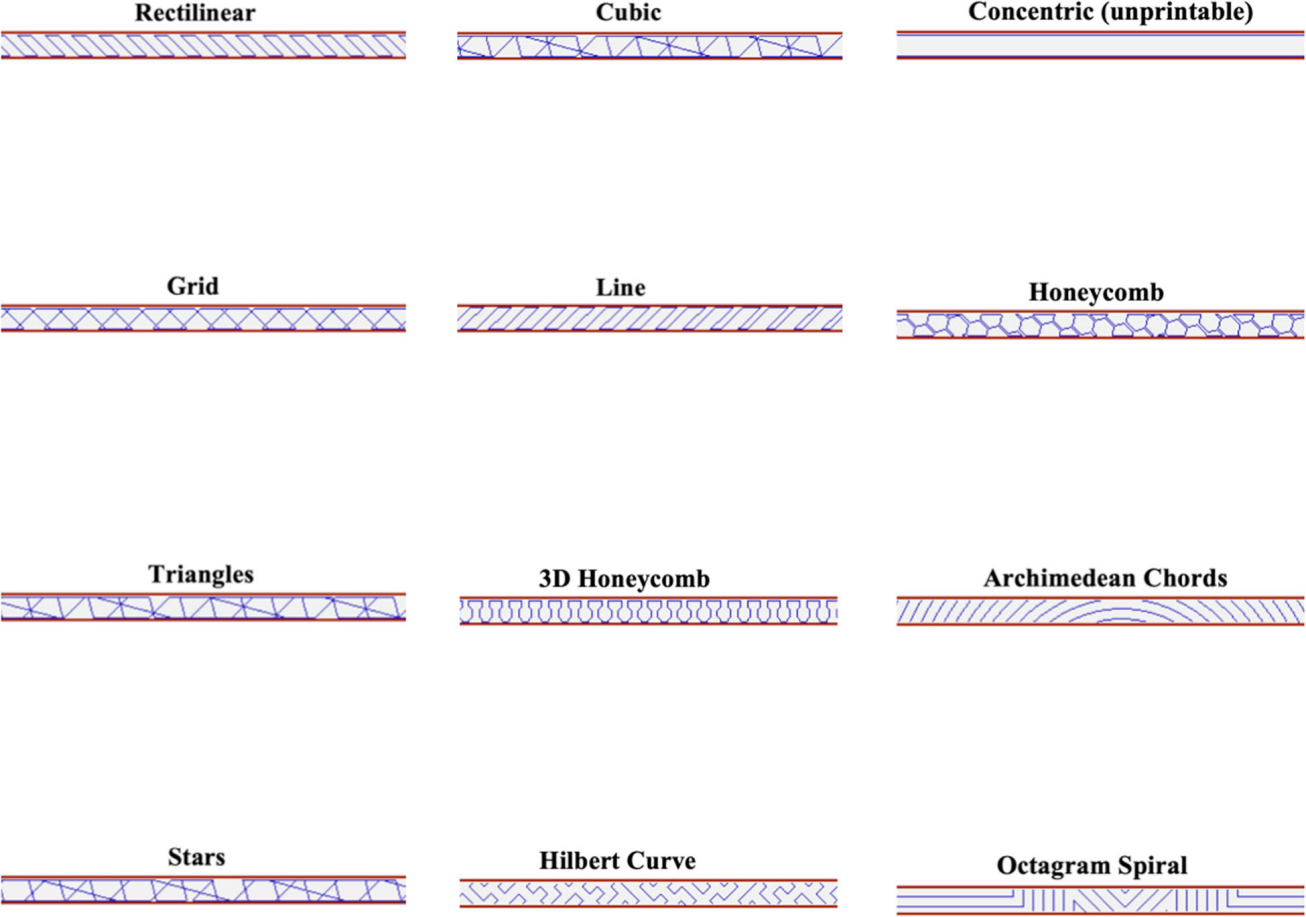
Retractor infill geometries

To test whether infill geometry affects retractor strength, a single 20% infill, non-reinforced, 0.25 in. thickness, 0.5 in. width, and 2 perimeter Essentium filament retractor was printed for eleven different geometries: Rectilinear, Grid (default), Triangles, Stars, Cubic, Line, Honeycomb, 3D Honeycomb, Hilbert Curve, Archimedean Chords, and Octagram Spiral (Fig. 3). The “Concentric” geometry was found to be unprintable due to the narrowness of the retractor. The time to print these retractors ranged from 42 min (Rectilinear) to 1 h 20 min (Grid) depending on the complexity of the infill. The following infill geometries had a print time of less than 50 min: Rectilinear, Triangle, Stars, Cubic, and Line. The following infill geometries had a print time of more than 1 h: Honeycomb, 3D Honeycomb, Hilbert Curve, Archimedian Chords, Octagram Spiral, and Grid.

These retractors were broken using a Nidec-Shimpo FGS-1000H hand wheel test stand and FG-3009 force gauge. From this data, the two strongest retractor geometries, “Cubic” and “Triangle”, and two weakest retractor geometries, “3D Honeycomb” and “Grid”, were studied further. For each of the geometries “Cubic”, “Triangle”, and “3D Honeycomb”, twelve retractors were printed. For the “Grid” geometry, retractor data from sets number 1, 2, and 4 were combined to create an aggregate Essentium filament control set and used as comparison.

To test how infill percent affects retractor strength, sets of non-reinforced, 0.25 in. thickness, 0.5 in. width, 2 perimeter Essentium filament retractors were printed at various infill percentages (Table 2). For 20% infill, retractors from the aggregate Essentium filament control set were used. The estimated mass of the retractors varied from 8.05 g to 27.39 g, resulting in a material cost of $0.40 to $1.37 USD without machine expenses. The time to print these retractors ranged from 40 min to 1 h 40 min, increasing as infill percentage increased.

**Table 2:** Retractors varied by infill percentage

Number of Retractors	Infill Percentage	Estimated Mass (g)	Estimated Cost (USD)
4	10%	8.05	$0.40
22	20%	10.20	$0.51
4	60%	18.79	$0.94
4	80%	23.09	$1.15
4	100%	27.39	$1.37

To test how perimeter number affects retractor strength, sets of 20% infill, non-reinforced, 0.25 in. thickness, and 0.5 in. width Essentium filament retractors were printed at various perimeter numbers (Table 3). Each perimeter is 0.15 mm. For 2 perimeter retractors, retractors from the aggregate Essentium filament control set were used. The estimated mass of the retractors varied from 10.20 g to 17.27 g, resulting in a material cost of $0.51 to $0.86 USD without machine expenses. The time to print these retractors ranged from 47 min to 54 min, increasing as perimeter number increased.

**Table 3:** Retractors varied by perimeter number

Number of Retractors	Perimeter Number	Estimated Mass (g)	Estimated Cost (USD)
22	2	10.20	$0.51
4	3	12.55	$0.63
4	4	14.91	$0.75
4	5	17.27	$0.86

To test how width affects retractor strength, sets of 20% infill, non-reinforced, 0.25 in. thickness, and 2 perimeter Essentium filament retractors were printed at various widths (Table 4). For 0.5 in. width retractors, retractors from the aggregate Essentium filament control set were used. The estimated mass of the retractors varied from 10.20 g to 20.39 g, resulting in a material cost of $0.51 to $1.02 USD without machine expenses. The time to print these retractors ranged from 1 h 28 min to 2 h 35 min, increasing as width increased.

**Table 4:** Retractors varied by width (inch)

Number of Retractors	Width (in.)	Estimated Mass (g)	Estimated Cost (USD)
22	0.5	10.20	$0.51
4	0.75	15.29	$0.76
4	1.0	20.39	$1.02

To test how thickness affects retractor strength at non-100% infill percentages, sets of 20% infill, non-reinforced, 0.5 in. width and 2 perimeter Essentium filament retractors were printed at various thicknesses (Table 5). For 0.25 in. thickness retractors, retractors from the aggregate Essentium filament control set were used. The estimated mass of the retractors varied from 8.08 g to 10.20 g, resulting in a material cost of $0.40 to $0.51 USD without machine expenses. The time to print these retractors ranged from 56 min to 1 h 20 min, increasing as thickness increased.

**Table 5:** Retractors varied by thickness (inch) at 20% infill

Number of Retractors	Thickness (in.) at 20% In-fill	Estimated Mass (g)	Estimated Cost (USD)
4	0.15	8.08	$0.40
4	0.2	9.13	$0.46
22	0.25	10.29	$0.51

To further test how thickness affects retractor strength at 100% infill, sets of 100% infill, non-reinforced, 0.5 in. width and 2 perimeter Essentium filament retractors were printed at various thicknesses (Table 6). The estimated mass of the retractors varied from 16.80 g to 27.39 g, resulting in a material cost of $0.84 to $1.37 USD without machine expenses. The time to print these retractors ranged from 1 h 50 min to 2 h 4 min, increasing as thickness increased.

**Table 6:** Retractors varied by thickness (inch) at 100% infill

Number of Retractors	Thickness (in.) at 100% In-fill	Estimated Mass (g)	Estimated Cost (USD)
4	0.15	16.80	$0.84
4	0.2	22.07	$1.10
4	0.25	27.39	$1.37

To test how reinforced joints affects retractor strength, sets of 40% infill, 0.25 in. thickness, 1 in. width, and 2 perimeter Essentium filament retractors were printed either reinforced or non-reinforced. 3 retractors were printed non-reinforced and 4 retractors were printed reinforced. The estimated mass of a non-reinforced and reinforced retractor, respectively, is 28.99 g and 32.78 g, resulting in a material cost of $1.45 and $1.64 USD without machine expenses. The time to print these retractors ranged from 2 h 31 min (non-reinforced) to 2 h 43 min (reinforced).

## Results

### Inter-spool and inter-manufacturer variability

**Fig. 4:**
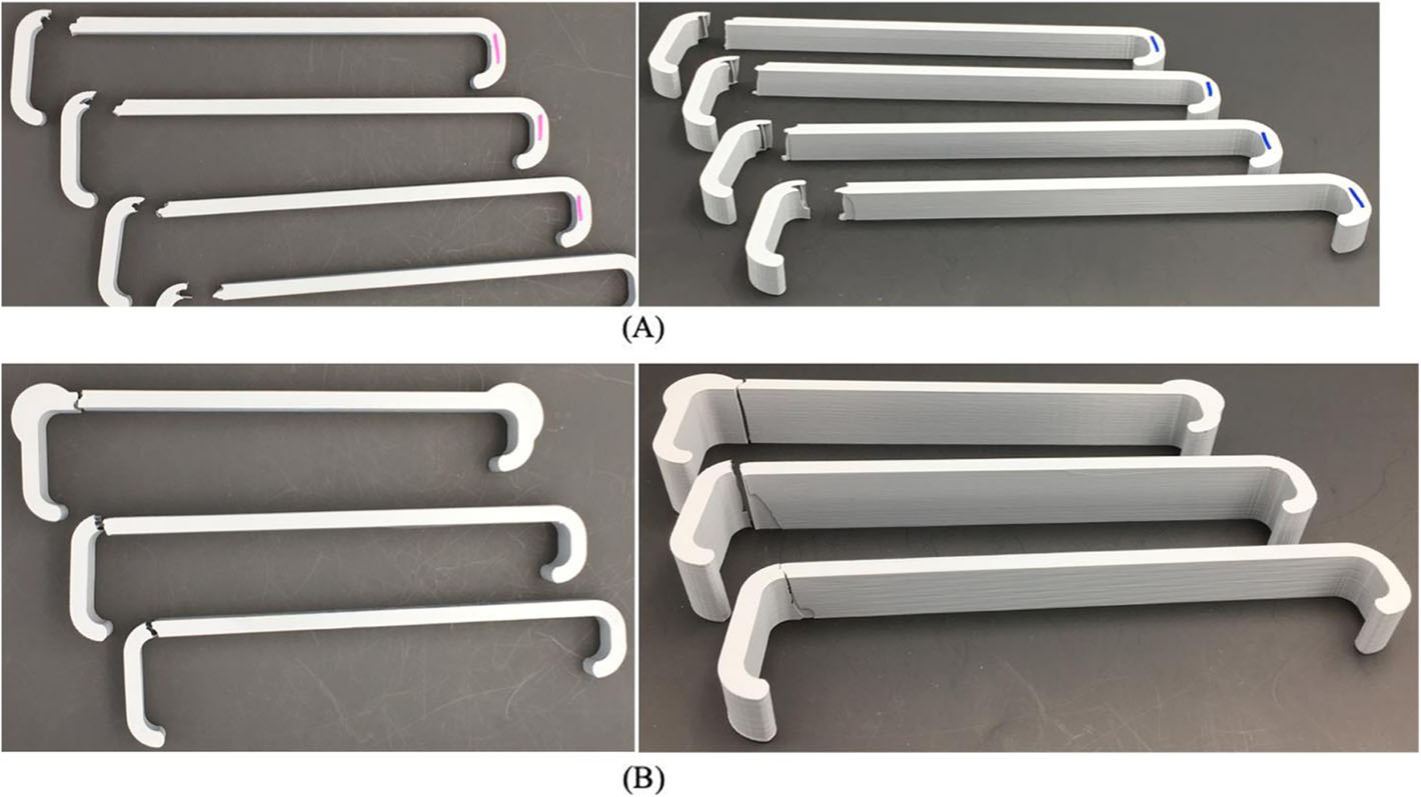
**a** Broken standard retractors and **b** broken retractors with (top to bottom): 1 in. width with reinforced joints, 1 in. width, and 0.75 in. width

Essentium filament control retractors failed at a load of 90.7 N +/− 3.3 N. MakerBot filament control retractors failed at a load of 108.6 N +/− 9.5 N. All retractors in this study failed at the joint connecting the body of the retractor to the long arm (Fig. 4).

**Fig. 5:**
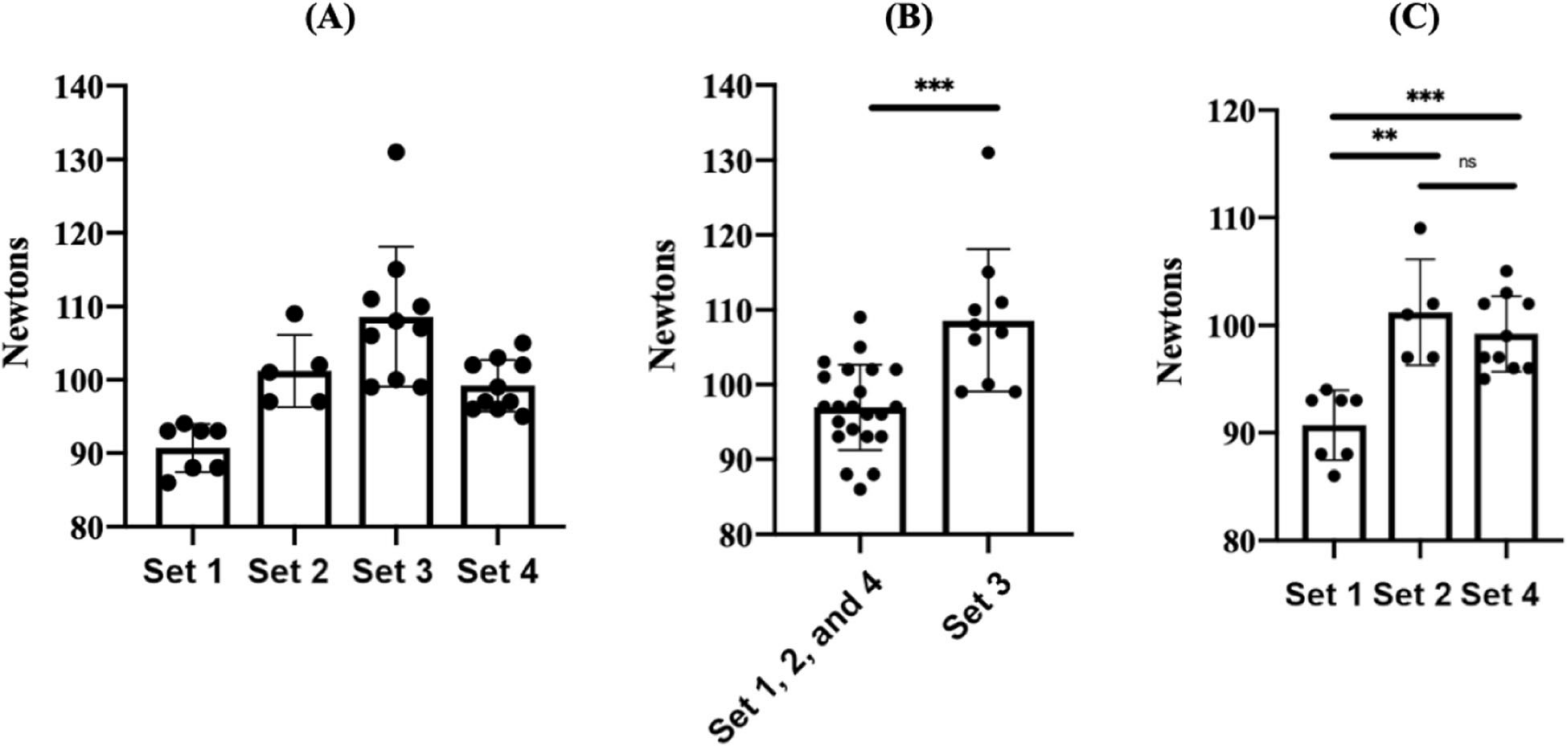
**a** Interspool and inter manufacturer variability affects control retractor strength. **b** Intermanufacturer variability affects control retractor strength. **c** Interspool variability affects control retractor strength

Using a two-tailed T-test, the strength of retractors in Set 1 (Essentium PLA spool 1) was found to be statistically different than Sets 2 (Essentium PLA spool 2, *p*-value = 0.0012) and 4 (Essentium PLA spool 3, *p*-value = 0.0001); Sets 2 and 4 were not statistically different (*p*-value = 0.3783) (Fig. 5). The Essentium filament set, an aggregation of Sets 1, 2, and 4, was statistically different than the MakerBot PLA set, set 3 (MakerBot PLA, *p*-value = 0.0002) (Fig. 5). The data demonstrates statistically significant differences in retractor strength between spools of the same manufacturer and between manufacturers.

### Optimizing retractor parameters

**Fig. 6:**
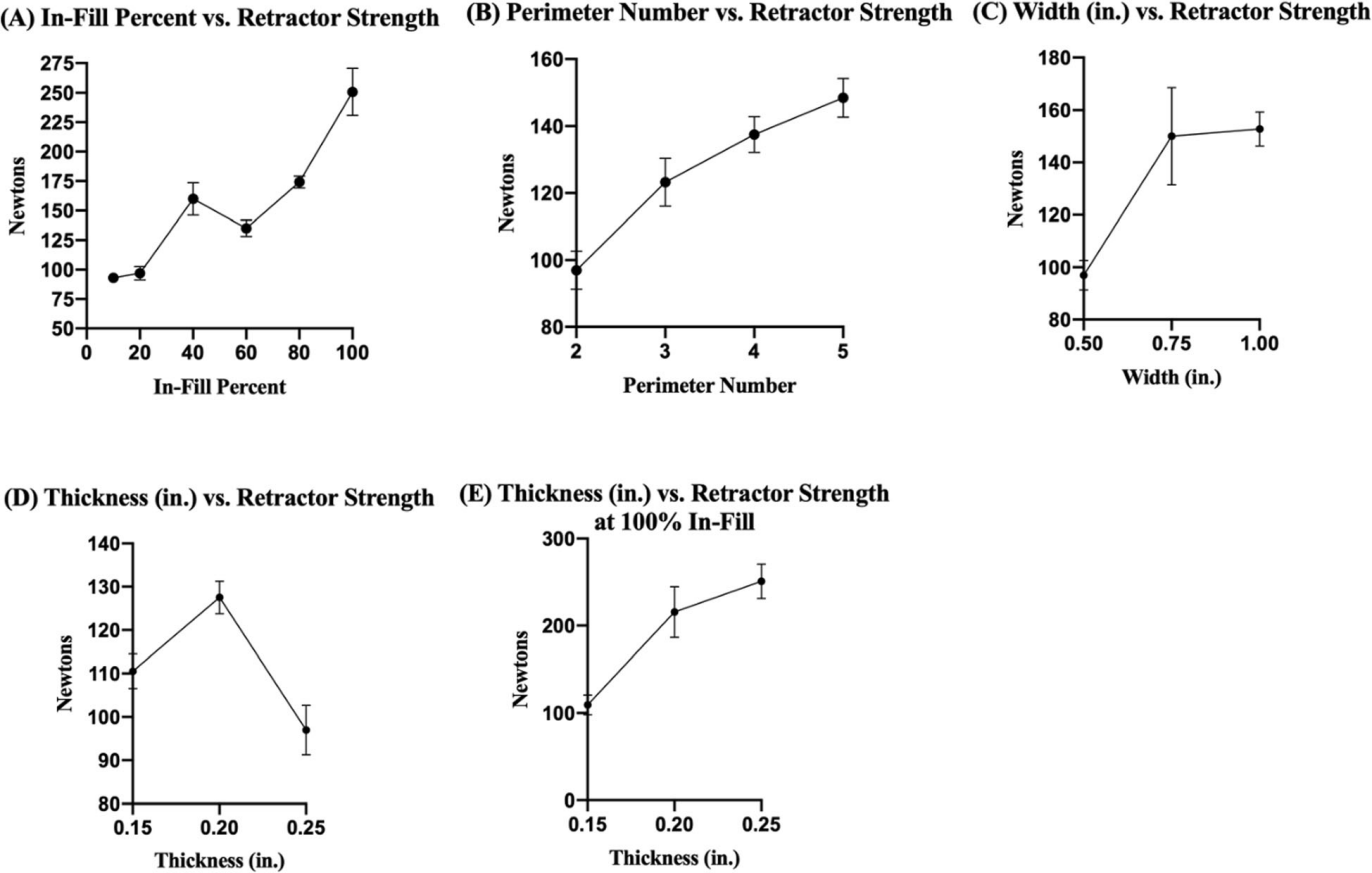
**a** As infill percent increases, retractor strength increases with a spike at 40%. **b** As perimeter number increases, retractor strength increases. **c** As retractor width increases, retractor strength increases up to 0.75 in. **d** Retractor thickness is most optimal at 0.2 in. at 20% infill. **e** Increasing retractor thickness increases retractor strength at 100% infill

As infill percent increased, retractor strength generally increased at 2 perimeters, with a spike at 40% infill. As predicted, 10% infill resulted in the weakest retractor, and 100% infill resulted in the strongest retractor. 40% and 80% infill resulted in retractors with comparable strengths (Fig. 6a). As perimeter number increased, retractor strength increased at 20% infill (Fig. 6b). As retractor width increased, retractor strength increased up to 0.75 in. then plateaued up to 1 in. (Fig. 6c). At 20% infill, retractor thickness was most optimal at 0.2 in. (Fig. 6d). At 100% infill, retractor strength increased as retractor thickness increased (Fig. 6e).

A 1 in. width retractor at 40% infill failed at 330.3 N +/− 2.1 N. A 1 in. width retractor at 40% infill with reinforced joints failed at 351.8 N +/− 6.1 N. At 20% infill, the two strongest geometries were “Cubic” and “Triangles”, which failed at 170.0 N +/− 4.8 N and 178.2 N +/− 4.2 N respectively; the two weakest geometries were the “3D Honeycomb” (99.0 N +/− 8.9 N) and “Grid” (97.0 N +/− 5.7 N).

## Discussion

### Retractor efficacy

All printed retractors in this optimization study have exceeded the threshold for clinically excessive retraction of 35 N by a large margin, at minimum by 2X and at maximum by 15X after determining optimal infill geometry, infill percentage, perimeter number, width, thickness, and design at the joints. Above 35 N, tissue injury becomes a clinical concern. Our control retractors, printed with default settings on average failed at almost 3X the load and the strongest retractors required almost 15X the load.

Generally, higher infill percentages, increased thickness, increased width, increased perimeter number and reinforced joints each lead to increased strength, print times, and material use. Depending on the availability of resources and time, print settings can be adjusted to create a sufficiently strong retractor while reducing cost, material use, and print time. The adaptability of 3D printing to various situations in medicine is a major strength and reason to adopt this technology. This is further accompanied by the fact that 3D prints are sterilized upon heating and extruding, allowing them to be used in sterile fields, and that they are substantially lower cost than traditional stainless steel retractors [21, 22].

Because retractor strength was greatest at 0.2 in. thickness for 20% infill retractors and retractor strength increased as retractor thickness increased for 100% infill retractors, we are led to conclude that the flexibility of retractors delay mechanical failure by reducing strain on the joints, the reliable point of failure. We hypothesize that the spike in retractor strength at 40% infill is also because of the bend and flex of the retractor reducing strain on the joints.

Regarding printing time, increasing perimeter number, increasing thickness, and adding reinforced joints did not substantially increase printing time. However, increasing width and infill percentage resulted in a marked increase in printing time, the former due to the addition of more layers and the latter increasing the time to build each layer.

### True optimization and limitations

A retractor that is optimized to the greatest theoretical possible strength given the parameters in this study has 100% infill, 5 perimeters, 0.25 in. thickness, 1 in. width, and has reinforced joints. However, upon printing, it was discovered that this retractor was unprintable. This study revealed that 3D prints have a higher rate of failure as infill percentage increases. We believe that this may be due to the printer slicer software miscalculating the volume of plastic extruded, thereby attempting to fit greater amount of plastic in a layer than can actually fit. This then causes the plastic to overflow into upper layers, causing rough and uneven prints that may be pushed off the build plate by the extruder. Therefore, incrementally reducing infill percentage, perimeter number, and maintaining a 0.25 in. thickness in order to accommodate more plastic, we determined the true strength optimized retractor to have 30% infill, 3 perimeters, 0.25 in. thickness, 0.75 in. width, and has “Triangle” infill geometry and reinforced joints. This retractor fails at 534 N +/− 28.5 N, more than 15X the threshold for clinically excessive retraction, 35 N. The estimated mass of this retractor is 24.94 g, resulting in a material cost of $1.25 USD without machine expenses, only 5.2% the online retail price of a stainless steel Army-Navy retractor at $24 per unit. The time to print these retractors is approximately 2 h 14 min.

We acknowledge that even though interspool variability exists, our retractor sets were printed randomly using different spools. Even with this variability, we were still able to derive statistically significant differences in retractor strength as parameters were varied systematically.

## Conclusion and future steps

This study demonstrates how even pre-optimized 3D printed PLA Army-Navy retractors have comparable surgical retraction capabilities to stainless steel retractors, but can be created in-house at a fraction of the cost and adopted in resource poor settings. These retractors fail at a force that greatly exceeds what is required for clinically excessive retraction, 35 N. The optimization of 3D printed Army-Navy retractors greatly improve the efficacy of this instrument and expedite the adoption of 3D technology in many diverse fields in medicine not necessarily limited to resource-poor settings. With the optimization of the 3D printed PLA Army-Navy retractor, a staple surgical instrument, researchers can pave the way towards and gain trust for an array of 3D printed medical supplies, allowing populations around the globe to receive higher quality health care and treatment.

A quantitative optimization of 3D printed Army-Navy retractors reveal their surgical capabilities to be comparable to commercial stainless steel retractors, but can be created in-house at a fraction of the cost and can be rapidly prototyped. This allows resource scarce areas to expand their access to high-quality medical equipment and direct their funds towards additional medical and social needs, improving healthcare around the world. Research on 3D printed surgical instruments opens additional discussion about the efficacy of 3D printed surgical tools and sparks a new field of research in 3D print optimization and dissemination.

Surgical use of these instruments, which are FDA class I devices, will require additional biocompatibility and sterilization validation. For medical use, this validation is done on the device level and not on the materials level. We know that PLA as a source material is biocompatible, but additives that are added to the PLA for coloring or for printability, may not be biocompatible [23]. There is data that supports that the fused deposition modeling printing process, where the thermoplastics are heated to above 200 °C, in our case 235 °C then extruded at high pressure through a 0.4 mm nozzle is in fact a sterilization process [21]. We have data from our lab that shows that post-processing of PLA prints can in fact be processed with steam sterilization, despite prior data that suggest that it would not be possible [24]. This is due to advances in PLA polymer technology and the ability to anneal and crystallize the PLA device after printing. This manuscript is intended as a pre-clinical evaluation and optimization of 3D printing technology. Additional research will be needed to assess performance after steam sterilization, printing with different nozzle diameters, and with different materials.

## Data Availability

Retractor STLs and data are available through the authors.

## Abbreviations

PLA: Polylactic Acid
STL: Standard Tessellation Language
